# The role of exercise in the treatment and recovery process of anorexia nervosa

**DOI:** 10.1186/2050-2974-1-S1-O8

**Published:** 2013-11-14

**Authors:** Sarah Young, Paul Rhodes, Stephen Touyz, Phillipa Hay

**Affiliations:** 1 The University of Sydney, Australia; 2 University of Western Sydney, Australia

## 

The detrimental role of excessive exercise in the pathogenesis and maintenance of Anorexia Nervosa (AN) has featured in past research (Casper, 1998; Davis, 1997). A scarcity of research has focused on targeted exercise interventions in treatment and recovery. Research indicates eliminating exercise completely during treatment is not therapeutic (Beumont, Arthur, Russell & Touyz, 1994), and exercise interventions can be beneficial for improving psychological outcomes (Hausenblas, Cook & Chittester, 2008). The current study aims to investigate the role of exercise in treatment and recovery. 24 participants (12 currently diagnosed with AN, 12 recovered from AN) complete the Eating Disorder Examination (Fairburn, Cooper & O'Connor, 2008), Compulsive Exercise Test (Taranis, Touyz & Meyer, 2011) and a semi-structured interview assessing exercise attitudes and behaviours across their lifespan (including through AN). Data collection is ongoing, with interview data analysed qualitatively using narrative inquiry and grounded theory methods. Preliminary data suggests that for some participants, exercise played a pivotal role in treatment and recovery. Thematically, it appears there is a subgroup of participants for whom exercise was a part of their identity pre-morbidly, and that re-establishing healthy exercise is an integral part of their recovery process. Implications for clinical treatment options will be discussed.

This abstract was presented in the **Adult Treatment and Services** stream of the 2013 ANZAED Conference.

